# Extracellular vesicle-mediated ferroptosis, pyroptosis, and necroptosis: potential clinical applications in cancer therapy

**DOI:** 10.1038/s41420-024-01799-6

**Published:** 2024-01-12

**Authors:** Yi-Chi Yang, Qian Jiang, Ke-Ping Yang, Lingzhi Wang, Gautam Sethi, Zhaowu Ma

**Affiliations:** 1 School of Basic Medicine, Yangtze University, Health Science Center, Yangtze University, 434023 Jingzhou, Hubei China; 2 Honghu Hospital of Traditional Chinese Medicine, 433200 Honghu, China; 3 Digestive Disease Research Institution of Yangtze University, Yangtze University, 434023 Jingzhou, China; 4 Department of Cardiology, Jingzhou Hospital Affiliated to Yangtze University, 434023 Jingzhou, China; 5 Department of Pharmacology, Yong Loo Lin School of Medicine, National University of Singapore, Singapore, 117600 Singapore; 6 NUS Centre for Cancer Research (N2CR), National University of Singapore, Singapore, 117599 Singapore; 7 Cancer Science Institute of Singapore, National University of Singapore, Singapore, 117599 Singapore

**Keywords:** Cancer therapy, Cancer microenvironment

## Abstract

Extracellular vesicles (EVs) have gained increasing recognition as significant regulators of intercellular communication in various physiological and pathological processes. These vesicles play a pivotal role in cancer progression by facilitating the transfer of diverse cargoes, including lipids, proteins, and nucleic acids. Regulated cell death (RCD), the orderly and autonomous death of cells, is controlled by a variety of biomacromolecules and, in turn, influences various biological processes and cancer progression. Recent studies have demonstrated that EV cargoes regulate diverse oncogenes and tumor suppressors to mediate different nonapoptotic forms of RCD, notably ferroptosis, pyroptosis, and necroptosis. Nevertheless, comprehensive exploration of EV-mediated nonapoptotic RCD forms in the context of cancer has not been performed. This review summarizes the progress regarding the biological functions and underlying mechanisms of EVs in mediating nonapoptotic RCD by delivery of cargoes to regulate tumor progression. Additionally, the review delves into the potential clinical applications of EV-mediated cell death and its significance in the areas of cancer diagnosis and therapy.

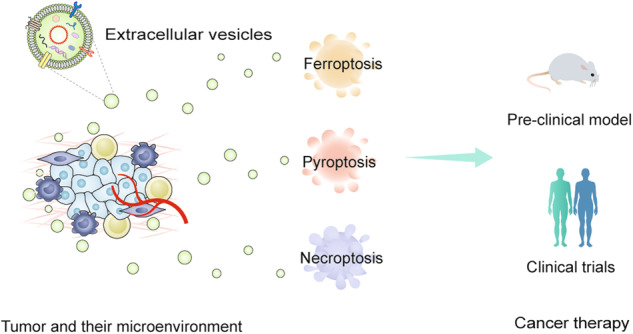

## Facts

- Extracellular vesicles (EVs) play a role in numerous pathophysiological processes, including nonapoptotic RCD.
- EVs act as a double-edged sword in tumor progression by mediating various forms of nonapoptotic RCDs, including ferroptosis, pyroptosis, and necroptosis.
- Targeting the various cargoes associated with EV-mediated cell death holds promise for advancing cancer therapy.

## Open questions

- What are the shared characteristics and distinctions between ferroptosis, pyroptosis, and necroptosis?
- What is the association between extracellular vesicle (EV)-mediated cell death and the response to cancer therapy?
- In what manner do EV-based drug delivery systems influence RCD processes and contribute to their anticancer efficacy?

## Introduction

Extracellular vesicles (EVs), including small EVs (sEVs) and large EVs(lEVs), are minute membrane-bound particles released naturally from cells and delimited by a lipid bilayer [1]. EVs can regulate intercellular communication by delivering numerous cargoes, comprising nucleic acids, lipids, and proteins, from donor to recipient cells, thereby contributing to multifarious physiological and pathological processes [2]. EVs derived from both normal and cancer cells can respond to extracellular and intracellular stress, including pH imbalance, platelet activation, ionizing radiation, low oxygen levels, radiation, chemotherapy, and necrosis. In response to a stress factor, cells can support the evasion of cell death within the tumor microenvironment (TME) while concurrently transmitting pro-survival information via EV-mediated intercellular communication to facilitate facilitating resistance to therapy [3]. Furthermore, EVs can influence the development and progression of cancer and are involved in inflammatory responses, metastasis, angiogenesis, epithelial-mesenchymal transition, invasion, cell migration, and proliferation [4]. EVs can also deliver a series of molecules to affect cell death in response to cellular stress stimuli [5]. For instance, a recent study revealed that sEVs can inhibit ferroptosis by facilitating the removal of iron from cells, thereby preventing cell death in the context of tumor suppression [6].

Cell death can be categorized as regulated cell death (RCD) and accidental cell death (ACD), depending on the morphology, biochemistry, and function [7]. ACD refers to cell suicide following injury. In contrast, RCD is regulated by controlled signaling pathways and contributes to disease development and maintenance of homeostasis [8]. RCD can be further subdivided into apoptotic and nonapoptotic types [9, 10]. Historically, apoptosis has been deemed the major form of RCD. However, contemporary research indicates that nonapoptotic RCD forms have not received adequate attention in the context of tumor cell biology and the mechanisms underlying cancer therapy [11, 12]. During the current decade, the most commonly studied types of non-apoptotic RCD have included ferroptosis, necroptosis, and pyroptosis [8, 13]. Furthermore, nonapoptotic RCD is closely associated with cancer development and the response to therapeutic interventions. In particular, nonapoptotic forms of RCD exhibit synergistic antitumor immune responses while possibly exerting inhibitory effects on tumor immune responses [14].

The regulation of nonapoptotic RCD by EVs provides a novel and promising strategy for addressing human diseases [15]. However, it is important to note that a comprehensive and systematic summary of nonapoptotic RCD mediated by EVs in cancer progression is currently lacking. In this context, our review aims to bridge this gap by exploring the roles of EVs in mediating ferroptosis, pyroptosis, and necroptosis across various cancer types. We believe that this exploration holds significant promise for clinical applications in the areas of cancer diagnosis and therapy.

## Overview of EVs

EVs are heterogeneous membrane vesicles that are actively secreted by virtually all types of cells, which can be released into the extracellular environment and serve as key mediators of intercellular communication [16–18]. Based on biogenesis, release pathways, subcellular origin, and size, EVs can be roughly classified into three types: microvesicles (MVs) with diameters of approximately 150–1,000 nm, exosomes with diameters of 50–150 nm, and apoptotic bodies with diameters ranging between 1–5 μm [1, 17, 19]. Exosomes are produced by the endosomal pathway through the endosomal sorting complex required for transport (ESCRT) mechanism, which contributes to the fusion of multivesicular bodies containing invaginated intraluminal vesicles with the plasma membrane and subsequent release into the extracellular space [17]. MVs, also referred to as shed MVs, are directly produced through outward budding and subsequent division of the cell membrane [20]. Apoptotic bodies, representing the largest subgroup of EVs, are generated via the apoptotic cell membrane during programmed cell death and can be phagocytosed by macrophages [21]. However, exosomes and MVs can be internalized through endocytosis [17]. Under physiological and pathological conditions, these internalized EVs can release intraluminal contents within recipient cells to mediate intercellular communication [22].

EVs also selectively deliver various cargoes from donor cells to recipient cells, such as proteins, nucleic acids, and lipids [23, 24], which can mediate different diseases by influencing a variety of signaling pathways [2]. EVs are emerging as excellent liquid biopsy analytes because they can be stably detected in various bodily fluids, such as serum, plasma, and urine [25]. Furthermore, the cargo carried by EVs can be used to assess the current disease status. Thus, EVs can be used as biomarkers for the diagnosis or detection of tumor progression [26]. It has been widely reported that EVs exert multiple effects to mediate cell migration and proliferation, angiogenesis, immune inflammation and modulation, evasion of cell death, tumor development, and metastasis [27, 28]. Furthermore, EVs, mainly exosomes and microparticles, participate in cancer progression and chemoresistance by mediating cell death signaling pathways [29–32].

## The main types of nonapoptotic RCDs

### Ferroptosis

Ferroptosis is an iron-dependent form of oxidative cell death characterized by oxidative modification [33]; it is distinct from apoptosis in terms of its biochemical, genetic, and morphological characteristics [34]. Its morphological characteristics include cell swelling and plasma membrane rupture. Ferroptosis is a direct result of peroxidation (excessive oxidative destruction) of cell membrane-associated lipids. The process depends on reactive oxygen species (ROS), iron, and phospholipids containing polyunsaturated fatty acids (PUFAs) [35]. Ferroptosis can be induced primarily through two distinct pathways—the transporter-dependent (extrinsic) pathway and the enzyme-regulated (intrinsic) pathway [36]. Both of these pathways are closely linked via different subcellular organelles and a series of metabolic pathways [37]. The extrinsic pathway is primarily induced via suppression of system X_c_^−^ [33], which is a cellular membrane amino acid transporter responsible for exporting glutamate and importing cystine, thereby regulating glutathione (GSH) biosynthesis. The intrinsic pathway can be triggered by inhibiting the activity or expression of glutathione peroxidase 4 (GPX4) via small-molecule substances [38]. Moreover, susceptibility to ferroptosis may be influenced by other signaling pathways, including ferroptosis suppressor protein 1 (FSP1)–coenzyme Q10 [39]. A series of antagonists and agonists orchestrate ferroptosis initiation and regulation. Lipophilic antioxidants, iron chelators, arachidonate lipoxygenase inhibitors, and acyl-CoA synthetase long-chain family member 4 (ACSL4) inhibitors block ferroptotic cell death [36]. Therefore, inhibiting system X_c_^−^, inducing consumption of GSH, and inactivating GPX4 are potentially effective treatment strategies to induce ferroptosis in cancer cells.

### Pyroptosis

Pyroptosis, which is also referred to as inflammatory cell necrosis, is a novel type of nonapoptotic RCD. The main characteristics of pyroptosis include cell membrane pore formation, membrane rupture, and cell lysis, leading to the release of proinflammatory cytokines [10]. Pyroptosis is mainly utilized by cells in the innate immune system in response to pathogen-induced signals and cellular perturbations triggered by inflammasomes and executed by gasdermin (GSDM) proteins, predominantly GSDMD and GSDME [40, 41]. Pyroptosis is generally regulated via two primary pathways, i.e., the classical and nonclassical pyroptosis pathways. The nonclassical pathway of pyroptosis is induced by direct stimulation and oligomer formation of caspase 4, caspase 5 (in humans), and caspase 11 (in mice [42]) through the binding of their amino (N)-terminal caspase activation and recruitment domain to the gram-negative bacterial lipopolysaccharide [43]). In contrast, the classical pathway of GSDMD activation is regulated via the activation of caspase 1 through the inflammasome signaling platform, which is assembled in response to a plethora of signals, including homeostasis-altering molecular processes, pathogen-associated molecular patterns, and damage-associated molecular patterns (DAMPs) [44]. Caspase 1 can both cleave and activate cytokines such as interleukin (IL)-1β and IL-18 to induce formation of their mature structures, eventually resulting in inflammatory responses and pyroptosis [45].

### Necroptosis

Necroptosis, a form of nonapoptotic RCD and regulated necrosis, is marked by distinctive morphological characteristics such as rupture of the plasma membrane, swelling of organelles, and leakage of intracellular contents [46]. Necroptosis is primarily regulated by receptor-interacting protein kinase (RIPK) 1, RIPK3, and even mixed lineage kinase domain-like protein (MLKL). Ligands tend to bind to specific necroptotic receptors (e.g., tumor necrosis factor [TNF] receptor 1), thus inducing necroptosis by facilitating the binding and activities of their respective cytoplasmic adaptor proteins [47]. The oligomerization and translocation of phosphorylated MLKL produce pores in the cell membrane, and this signal transduction leads to the leakage of cellular contents, including cytokines, DAMPs, chemokines, and interferons, thereby provoking an inflammatory response [48]. Thus, necroptosis must be strictly regulated to maintain normal tissue homeostasis [49, 50]. Recently, the significance of necroptosis in cancer has been increasingly appreciated, and a better understanding of necroptotic processes may be of use for developing novel cancer therapy strategies.

## EV-mediated RCD forms in cancers

**Fig. 1:**
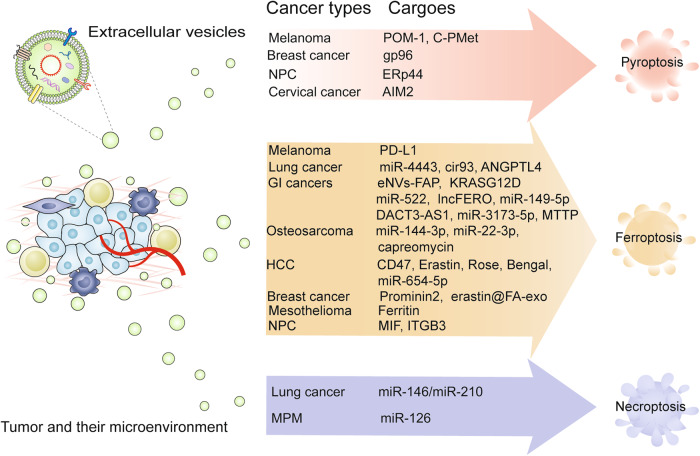
The emerging roles of EV-mediated non-apoptotic RCD in cancer. EVs transfer a wide range of key regulators to mediate diverse types of cell deaths, including ferroptosis, pyroptosis, and necroptosis, which promote or inhibit tumor development and progression. AIM2 absent in melanoma 2; ANGPTL4 angiopoietin-like 4; cir93 circRNA_101093; ERp44 endoplasmic reticulum resident protein 44; eNVs-FAP exosome-like nanovesicles fibroblast activation protein; FA folic acid; GI cancers gastrointestinal cancers; HCC hepatocellular cancer; ITGB3 integrin β3; MPM malignant pleural mesothelioma; MTTP microsomal triglyceride transfer protein; MIF macrophage migration inhibitory factor; NPC nasopharyngeal carcinoma; PD-L1 programmed cell death-ligand 1.

EVs mediate cancer development and propagation by influencing the crosstalk with local or remote recipient cells. Various cell-derived exosomes play crucial roles in establishing immune suppression, premetastatic niches, immune surveillance, immune escape, and maintenance of the tumor immune microenvironment [51]. EVs can induce cell-to-cell communication by transmitting intracellular contents to orchestrate cell death in numerous cancers [52, 53]. In the next sections, the expanding landscape of EV-mediated ferroptosis, pyroptosis, and necroptosis in diverse cancer phenotypes is discussed, and the related topics include drug resistance, tumor metastasis, and antitumor immunity (Fig. 1 and Table 1).

**Table 1:** The roles of EV-mediated ferroptosis, pyroptosis, and necroptosis in cancers.

Cancer type	Source cell	Cargo	Role in cell death	Functions	Ref.
*EVs in ferroptosis*					
Gastric cancer	CAFs	miR-522	Inhibit	Inhibits ferroptosis and enhances acquired chemoresistance	[29]
Gastric cancer	GC cell	lncFERO	Inhibit	Suppresses ferroptosis in GCSCs and reduces chemosensitivity of gastric tumors	[72]
Gastric cancer	CAFs	DACT3-AS1	Induce	Enhances the transformation of malignant masses and ferroptosis-mediated resistance to oxaliplatin in gastric cancer	[73]
Gastric cancer	Human umbilical cord-derived adipose-derived mesenchymal stem cells	miR-149-5p and siRNA	Induce	Enhances GC cells ferroptosis via inhibition of MKL-1 expression	[110]
Non-small cell lung cancer	NSCLC tumor tissue	miR-4443	Inhibit	Promotes resistance to cisplatin by regulating FSP1 m6A	[58]
Non-small cell lung cancer	NSCLC cell	ANGPTL4	Inhibit	Angiopoietin-like 4-mediated radioresistance of lung cancer by suppressing ferroptosis under hypoxicmicroenvironment	[67]
Lung adenocarcinoma	LUAD cell	circRNA_101093 (cir93)	Inhibit	Specifically desensitizes LUAD cells to ferroptosis and attenuates lipid peroxidation	[59]
Colorectal Cancer	Adipocyte	MTTP	Inhibit	Inhibits ferroptosis and promotes chemoresistance in colorectal cancer	[77]
Breast cancer	Breast carcinoma cells	Prominin2	Inhibit	Drives ferroptosis resistance	[6]
Triple-negative breast cancer	HFL‐1 cells	erastin@FA-exo	Induce	Inhibits expression of glutathione peroxidase 4 and upregulation of cysteine dioxygenase	[108]
Mesothelioma	Macrophage	Ferritin	Induce	Promotes asbestos-induced mesothelial carcinogenesis	[86]
Pancreatic ductal adenocarcinoma	PDAC cells	KRAS^G12D^	Induce	Inhibits of the release of KRAS^G12D^ from tumor cells	[117]
Pancreatic ductal adenocarcinoma	CAFs	miR-3173-5p	Inhibit	CAFs inhibit ferroptosis and induce gemcitabine resistance in pancreatic cancer cells by secreting exosome-derived ACSL4-targeting miR-3173-5p	[74]
Nasopharyngeal carcinoma	NPC cells	MIF	Inhibit	Promotes metastasis by inhibiting the ferroptosis of macrophages	[80]
Nasopharyngeal carcinoma	Platelet	ITGB3	Inhibit	Inhibits ferroptosis and enhances distant metastasis of nasopharyngeal carcinoma	[82]
Osteosarcoma	Osteosarcoma tissue	miR-144-3p	Induce	Promotes ferroptosis to inhibit osteosarcoma progression via the miR-144-3p/ZEB1 axis	[84]
Osteosarcoma	Cardiomyocytes	miR-22-3p	Induce	Aggravating tumor growth by suppressing the susceptibility to ferroptosis activation in myocardial infarction	[83]
Osteosarcoma	Bone marrow mesenchymal stem cells	capreomycin	Induce	Induced ferroptosis of osteosarcoma cells via the Keap1/Nrf2/GPX4 axis	[111]
Melanoma	B16F10 tumors	PD-L1	Induce	Enhances ferroptosis for synergistic immunotherapy	[103]
Melanoma	Melanoma cells	PD-L1	Induce	Reverses immune suppression and enhances ferroptosis	[102]
Hepatocellular cancer	HEK293T	CD47, Erastin, and Rose Bengal	Induce	Induces obvious ferroptosis in HCC with minimized toxicity in the liver and kidney	[109]
Hepatocellular cancer	Human adipose mesenchymal stem cells	miR-654-5p	Induce	Enhances sorafenib-induced ferroptosis of HCC cells via the miR-654-5p/HSPB1 axis	[112]
Colon, lung, melanoma, and breast cancer	Tumor cells	FAP gene-engineered (eNVs-FAP)	Induce	eNVs-FAP vaccine-induced immune responses could stimulate tumor ferroptosis by releasing IFN-γ from cytotoxic T lymphocyte and decreasing the levels of FAP +CAFs	[114]
*EVs in pyroptosis*					
Melanoma	B16F10 cells	POM1 and C-PMet	Induce	Remodels energy metabolism for initiating the adaptive and innate immune systems	[115]
Cervical cancer	SiHa cells	AIM2 inflammasome proteins	Inhibit	SIRT1-knockdown-derived EVs inhibit the growth of cervical cancer xenografts through the activation of AIM2 inflammasome	[30]
Breast cancer	Paclitaxel-resistant breast cancer cells	gp96	Induce	Increases resistance to paclitaxel and promotes immune evasion in breast cancer	[91]
Nasopharyngeal carcinoma	ER-stressed cells	ERp44	Inhibit	Strengthens cisplatin resistance of nasopharyngeal carcinoma	[92]
*EVs in necroptosis*					
Lung cancer	KrasLung tumor samples	miR-146/miR-210	Inhibit	Downregulates immunosuppressive BACH2/GATA-3 expression through RIP-3-dependent necroptosis and miR-146/miR-210 modulation	[31]
Malignant pleural mesothelioma	MPM cells	miR-126	Induce	miR-126 accumulation induces protective autophagy, and the suppression of this process by GW4869 gives rise to a metabolic crisis that favors necroptosis	[93]

*ACSL4* acyl-CoA synthetase long-chain family member 4, *ANGPTL4* angiopoietin-like 4, *AIM2* absent in melanoma 2, *CAFs* cancer-associated fibroblasts, *cir93* circRNA_101093, *EVs* extracellular vesicles, *ERp44* endoplasmic reticulum resident protein 44, *eNVs-FAP* exosome-like nanovesicles fibroblast activation protein, *GC* gastric cancer, *GCSCs* gastric cancer stem cells, *HCC* hepatocellular cancer, *IFN-γ* interferon gamma, *ITGB3* integrin β3, *LUAD* lung adenocarcinoma, *m6A* N6-methyladenosine, *MPM* malignant pleural mesothelioma, *MTTP* microsomal triglyceride transfer protein, *MIF* macrophage migration inhibitory factor, *NSCLC* non-small cell lung cancer, *NPC* nasopharyngeal carcinoma, *PDAC* pancreatic ductal adenocarcinoma cell, *PD-L1* programmed cell death-ligand 1, *RIP* receptor interacting protein, *SIRT1* sirtuin 1.

### The functions of EVs in ferroptosis

**Fig. 2:**
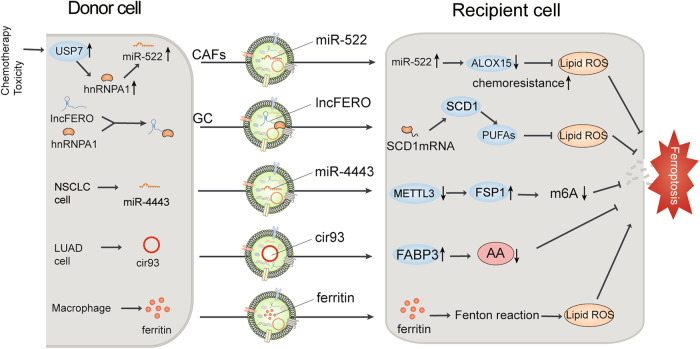
EVs transfer various cargoes to induce ferroptosis in cancer. Various cell-derived EVs transfer various cargoes (e.g., miR-522, lncFERO, ferritin) to recipient cells to promote or inhibit ferroptosis by affecting ferroptotic signal transduction in various cancers. AA arachidonic acid, ALOX15 arachidonate 15-lipoxygenase, CAFs cancer-associated fibroblasts, cir93 circRNA_101093, GC gastric cancer, FSP1 ferroptosis suppressor protein 1, FABP3 fatty acid-binding protein 3, PUFAs polyunsaturated fatty acids, hnRNPA1 heterogeneous nuclear ribonucleoprotein A1, m6A N6-methyladenosine, METTL3 methyltransferase-like 3, NSCLC non-small cell lung cancer, ROS reactive oxygen species, SCD1 stearoyl-CoA-desaturase 1, USP7 ubiquitin-specific protease 7.

Studies have demonstrated that EVs participate in and influence many cancer hallmarks, including resisting cell death, sustaining proliferative signaling, and activating invasion and metastasis [6, 54]. In the following section, we present the latest findings on the understanding of EV-mediated ferroptosis and its contribution to cancer chemoresistance, radioresistance, initiation, and progression (Fig. 2).

EV-mediated ferroptosis affects chemoresistance and radioresistance in lung cancer. In the case of non-small cell lung cancer (NSCLC), cisplatin is the first-line chemotherapeutic medicine that induces ferroptosis [55, 56]. However, cisplatin resistance has become increasingly common in NSCLC patients [57]. For instance, exosomal delivery of miR-4443 to cisplatin-sensitive NSCLC cells boosts FSP 1 expression while suppressing ferroptosis; thus, treatment of cisplatin-resistant NSCLC with antagomiR can restore responsiveness to cisplatin [58] (Fig. 2). Lung adenocarcinoma (LUAD)-derived exosomal circRNA_101093 (cir93) increases fatty acid-binding protein 3 (FABP3) to reduce global arachidonic acid through reactions with taurine, thus inducing ferroptosis by desensitizing LUAD cells and decreasing total lipid peroxidation. PDX mouse model data revealed that poor survival of LUAD and resistance to ferroptosis were predicted by elevated levels of FABP3 and cir93 [59] (Fig. 2). In addition to their effects on chemoresistance, exosomes can also affect radioresistance by regulating ferroptosis [29, 58]. To date, NSCLC treatment predominantly relies on surgery, chemotherapy, immunotherapy, radiotherapy, and local interventional therapy. Although radioresistance mechanisms have been explored extensively [60, 61], the five-year survival rate is <17% owing to tumor radioresistance [62]. The angiopoietin-like 4 (ANGPTL4) gene, an inflammatory carcinogenic regulator and a decisive angiogenesis mediator [63–65], has been identified as a key ferroptosis-related gene [66]. Under hypoxia, the ANGPTL4 protein can be transferred by exosomes to bystander normoxic NSCLC, thereby suppressing the onset of ferroptosis and promoting radioresistance in bystander cells [67].

In addition to those derived from cancer cells, sEVs derived from other cell types may influence chemoresistance in preclinical tumor models of gastric cancer (GC). For example, exosome-derived miR-522 from cancer-associated fibroblasts (CAFs) could block the accumulation of lipid ROS by targeting ALOX15 in GC cells, thereby inhibiting ferroptosis and inducing chemotherapy resistance [29] (Fig. 2). Chemotoxicity in CAFs increases the expression of ubiquitin-specific protease 7 (USP7), a promising target for antitumor drug resistance [68, 69]. Heterogeneous nuclear ribonucleoprotein A1 (hnRNPA1) is associated with the exosomal secretion of multiple miRNAs [70, 71]. USP7 enhances the secretion of miR-522 from CAFs by mediating hnRNPA1 deubiquitination. miR-522 levels significantly decrease in the tumor microenvironment due to the knockdown of USP7 or hnRNPA1, thus causing attenuated chemosensitivity and enhanced cell death [29] (Fig. 2). Furthermore, the pathway associated with hnRNPA1, involving stearoyl-CoA-desaturase 1 (SCD1) and ferroptosis-associated lncRNA (lncFERO), also exhibits exosome-ferroptosis effects. Chemotoxicity stimulates GC cells to secrete lncFERO through the upregulation of hnRNPA1 expression. Exo-lncFERO derived from GC cells then enters gastric cancer stem cells (GCSCs) and upregulates SCD1 expression in GCSCs by binding SCD1-associated mRNA and recruiting hnRNPA1, thereby suppressing GCSC ferroptosis and reducing chemosensitivity in GC cells. hnRNPA1 knockdown in GCSCs prevents this effect; thus, targeting the exo-lncFERO/hnRNPA1/SCD1 axis combined with chemotherapy might prove to be a promising CSC-based strategy for GC treatment [72] (Fig. 2). Another recent study demonstrated that CAF-derived exosomal DACT3-AS1, as a GC-associated suppressive regulator, sensitized GC cells to oxaliplatin treatment via Sirtuin 1 (SIRT1)-regulated ferroptosis conferring ferroptosis-mediated oxaliplatin sensitivity [73].

Exosomes derived from other cell types can also regulate chemoresistance in other digestive system tumors. After receiving chemotherapy with gemcitabine, CAFs can secrete exosomes with high expression of miR-3173-5p, which is internalized by pancreatic ductal adenocarcinoma (PDAC) cells. Mechanistically, exosome promote resistance to gemcitabine by suppressing ferroptosis via the miR-3173-5p/ACSL4 axis [74]. Colorectal cancer (CRC)-related adipokine secretion is linked to cancer progression and chemoresistance. Microsomal triglyceride transfer protein (MTTP), a major intracellular lipid transfer protein, is delivered by adipose-derived exosomes to inhibit ferroptosis in CRC cells. Mechanistically, this study revealed that exosomes promoted oxaliplatin resistance and inhibited ferroptosis by upregulating GPX4 and xCT in CRC organoids [75–77].

Emerging studies have demonstrated a role for sEV-mediated ferroptosis in different stages of tumor development. Metastasis is the leading contributor to resultant cancer mortality in patients [78]. Macrophages are one of the most common host immune cells in the TME and regulate metastasis [79]. Nasopharyngeal carcinoma (NPC) is the most prevalent malignant tumor among cancers of the head and neck. Macrophage migration inhibitory factor (MIF), an inflammatory cytokine, is positively correlated with poor prognosis in NPC patients. MIF is highly expressed in NPC cells, and their secreted exosomes can be absorbed by macrophages; MIF-rich exosomes can thus suppress ferroptosis in macrophages and thereby promote NPC metastasis [80]. Platelets are versatile cells that are part of the pathological processes of tumor cell hematogenous metastasis [81], and platelet-derived EVs from NPC patients upregulate integrin β3 (ITGB3) while elevating the expression of solute carrier family 7 member 11 (SLC7A11) by activating the MAPK/ERK/ATF4/Nrf2 axis and increasing protein stability. This process suppresses ferroptosis, thus facilitating the distant metastasis of NPC cells through blood circulation [82]. Interestingly, exosomes secreted by different sources mediate intercellular communication by transferring diverse cargoes, consequently exerting a discernible influence on cancer progression. Exosomal miR-22-3p is transferred from cardiomyocytes to osteosarcoma cells, thereby aggravating tumor growth by suppressing susceptibility to ferroptosis activation in myocardial infarction [83]. Osteosarcoma tissue-derived exosomes promote ferroptosis to inhibit osteosarcoma progression via the miR-144-3p/ZEB1 axis [84]. Asbestos-related diseases still remain a societal burden worldwide [85]. A remote, novel mutagenic mechanism of loading iron into mesothelial cells through ferroptosis-dependent EVs (FedEVs) containing ferritin was suggested. In this scenario, macrophages that engulf asbestos generate FedEVs, and ferroptotic macrophage-derived extracellular vesicles are loaded with a high level of ferritin and are received by mesothelial cells, leading to considerable oxidative DNA damage, such as 8-OHdG generation and double-strand breakage, which ultimately cause asbestos-induced mesothelial carcinogenesis [86].

### EV-mediated pyroptosis in cancers

EV-mediated pyroptosis contributes to the development of various diseases. For instance, an exosome-based drug delivery system targeting CD44 loaded with forsythiaside A fights disease progression by regulating NLRP3-dependent pyroptosis [87]. EV-mediated pyroptosis influences cancer progression, and EVs can transfer specific cargoes, including drugs and bioactive molecules, to target cells, leading to pyroptotic cell death and drug resistance (Fig. 1 and Table 1).

Accumulating evidence suggests that therapeutic resistance to nonapoptotic RCD in cancer is closely linked to exosomes and the TME, such as hypoxia [88, 89]. Resistance to paclitaxel is a significant challenge in treating breast cancer. Hence, paclitaxel is ineffective for breast cancer treatment, causing a worse prognosis and even recurrence in some breast cancer patients [90]. A recent study demonstrated that hypoxic stress facilitated exosomal gp96 production and enhanced resistance to paclitaxel in paclitaxel-sensitive breast cancer cells, transformed these cells into paclitaxel-resistant breast cancer cells, and thus initiated pyroptosis-induced cell death in CD8^+^ T cells to facilitate immune escape [91]. Another similar study indicated that exosomal endoplasmic reticulum resident protein 44 (ERp44) derived from endoplasmic reticulum-stressed cells promotes cisplatin resistance in nasopharyngeal carcinoma, thereby mediating cell apoptosis and pyroptosis [92].

Cargoes carried by exosomes mediating pyroptosis can be potentially be used as therapeutic targets for treating different types of cancer. SIRT1, a nicotinamide adenine dinucleotide-dependent deacetylase, may be a potential target for cervical cancer therapy. Since it is highly expressed in cervical cancer due to HPV infection, it is crucial for cervical cancer progression and is even linked to poor clinical outcomes. Furthermore, SIRT1 allows HPV-infected cervical cancer cells to maintain growth levels by nullifying absent in melanoma 2 (AIM2) inflammasome-mediated immunity, and silencing SIRT1 causes these cancer cells to undergo pyroptosis regulated by EVs carrying AIM2 inflammasome proteins. [30]. These findings indicate that cancer cell-derived EVs exert potent effects on tumor progression by inducing pyroptotic cell death, which suggests a promising approach for cancer therapy.

### EV-mediated necroptosis in cancers

Necroptosis has a dual effect of promoting and reducing tumor growth. Emerging evidence suggests that EV-mediated necroptosis can also control the migration, proliferation, and invasion of tumor cells. RIP1 and RIP3 are essential for necroptosis, and the complex mediates death receptor-dependent necroptosis [78], Exosomal miRNAs cause necroptosis by binding and regulating RIP3. For example, in chemoresistant tumors, cotreatment with Kras-derived exosomes (circulating Kras exosomes isolated from metastatic lung cancer patients) and carboplatin induced RIP3/TNF alpha-regulated necroptosis accompanied by miR-146/miR-210 modulation in patients with metastatic lung cancer [31]. Kras-derived exosomes offer new opportunities for inhibiting metastatic neoplasia by sustaining lung immunosuppressive metabolism. Another miRNA-based therapy combinated with GW4869, an inhibitor of exosome release, has been applied in malignant pleural mesothelioma (MPM). Treatment of MPM-derived spheroids with miR-126-enriched exosomes induced antitumor effects. Treatment with exosomes enriched in miR-126 plus the inhibitor of exosome release (GW4869) led to the accumulation of miR-126 inside cells, thereby promoting necroptotic activation of MPM-stem cells [93].

The protein content of necroptotic EVs was characterized by high-throughput proteomic analyses. A study employed TNF-dependent necroptosis and apoptosis in human primary macrophages and a lymphoma cell line. The supernatants and enriched EVs were also subjected to proteomic analysis, which revealed the cell death type-specific release of cytokines as well as the underlying processes regulated during apoptosis and necroptosis [94]. Proteomic analysis of necroptotic EVs revealed an additional regulatory mechanism during the early stage of necroptosis; this mechanism was mediated by specific EV cargoes, which reshape the tumor microenvironment and induce both adaptive and innate immune responses [95].

Further research is needed regarding the necroptotic pathways to establish their molecular mechanism as well as the relationship between downstream and upstream signaling molecules of cell death signaling pathways. This would also help researchers to explore its dual role in bilateral communication and to identify relevant targeted drugs to enhance the effect of tumor therapies.

## Targeting EV-regulated RCD for cancer therapy

**Fig. 3:**
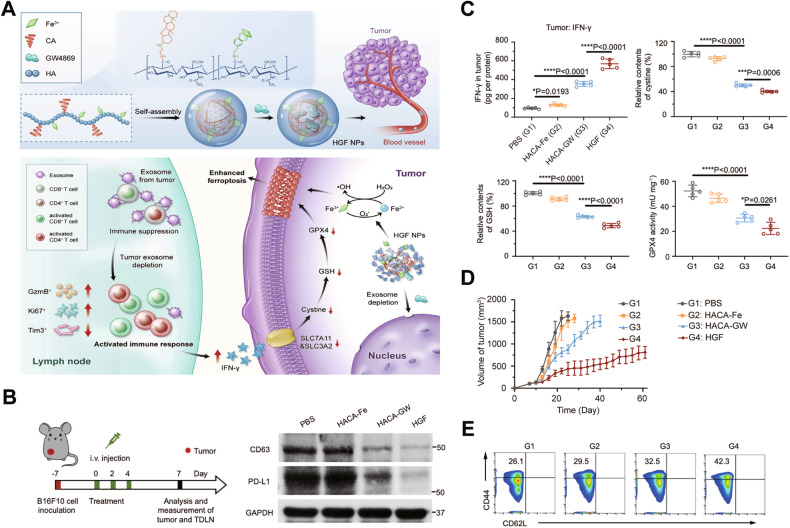
HGF NPs act with anti-exosomal PD-L1 to reverse immune suppression and enhance ferroptosis. **A** Schematic illustration of HGF-relevant preparation and therapeutic strategy. **B** Scheme of in vivo experiments and western blot analysis of the exosome markers CD63 and PD-L1 in tumor tissues after treatment. **C** IFN-γ level and relative levels of cystine (Cys)/GSH and GPX4 activity in tumors after treatment. **D** Tumor growth curves during treatment. **E** Flow cytometric analysis of memory T cells (CD44^high^CD62^low^, gating on CD3^+^CD8^+^ T cells) in the spleen. Adapted with permission from [102]; copyright 2021, Springer Nature.

Chemotherapy, radiotherapy, surgery, and immunotherapy are the main therapeutic strategies applied to tumor treatment. Therapeutic advances in cancer immunotherapy have rapidly emerged in the past few years [96]. The use of conventional therapy in conjunction with RCD modulators might hold significant potential for cancer treatment. EV-mediated ferroptosis is a part of tumor resistance and T-cell immunity [97–99]. Thus, the development of therapeutic strategies combining ferroptosis inducers with exosomal inhibitors is a promising avenue, and the combination of multiple drugs (e.g., GW4869, Fe^3+^) may enhance cancer immunotherapy effects. Complexes carrying tumor cell-derived exosomal PD-L1 (e.g., melanoma) suppress the activity of T cells and lead to resistance to tumor therapy [100, 101]. A hyaluronic acid-based nanoplatform (referred to as HGF NPs) was developed by combining a ferroptosis inducer (Fe^3+^) with an exosome inhibitor (GW4869) to induce antitumor responses in melanoma cells. GW4869 released from HGF NPs markedly inhibited tumor-derived exosome generation and exosomal PD-L1 and promoted T-cell activation. Furthermore, reactivated T cells release high levels of interferon-gamma (IFN-γ) to suppress the SLC7A11-GSH-GPX4 axis, thus facilitating the ferroptosis of melanoma cells [102] (Fig. 3). Subsequently, semiconductor polymer assemblies encapsulating Fe^3+^ (ferroptosis inducers) and GW4869 (to block exosomal PD-L1) were used to establish phototheranostic metal-phenolic networks (PFG MPNs). PFG MPNs elicit a joint photothermal treatment with exosome-dependent immunotherapy, revitalizing T cells by antagonizing exosomal PD-L1-regulated suppression and boosting ferroptosis in cancer cells to evoke strong antitumor immunity in melanoma cells [103]. These findings demonstrated that the combination of ferroptosis inducers and exosome inhibitors in tumor therapy strategies has considerable prospects for clinical application.

Some compounds or drugs, such as artesunate, erastin, lycorine, FINO2, and altretamine, stimulate the ferroptosis in tumor cells [104]. Transporting these drugs through EVs to induce ferroptosis may be more effective than free drugs. Recent studies have revealed that artificially engineered exosomes hold extensive therapeutic potential in several cancers [105–107]. For example, exosomes targeting ferroptosis might be modified via folic acid (FA) and then used for clinical applications. Such exosomes containing the ferroptosis inducer erastin (erastin@FA-exo) confer antitumor effects by targeting folate receptor-overexpressing triple-negative breast cancer cells. Compared to free erastin, erastin@FA-exo increased the rate of erastin uptake in MDA-MB-231 cells and initiated ferroptosis by suppressing system X_c_^−^ [108]. Exosomal targeting of ferroptosis in combination with photodynamic therapy and immune modification efficiently induces antitumor effects in hepatocellular cancer (HCC) cells [109]. Moreover, modified mesenchymal stem cell-derived exosomes induce ferroptosis in cancer cells by delivering several therapeutic agents including clinical drugs, specific siRNAs and miRNAs [110–112]. Engineered exosome-based treatment has also been employed in a clinical trial. EVs have been used as a vaccine adjuvant to trigger a powerful anticancer response and hence to enhance the effects of cancer immunotherapy [113]. Hu et al. developed fibroblast activation protein-α (FAP) gene–engineered tumor cell-derived exosome-like nanovesicles (eNVs-FAP) as tumor-based vaccines that inhibited tumor growth by remodeling the tumor microenvironment and promoting tumor cell ferroptosis [114].

**Fig. 4:**
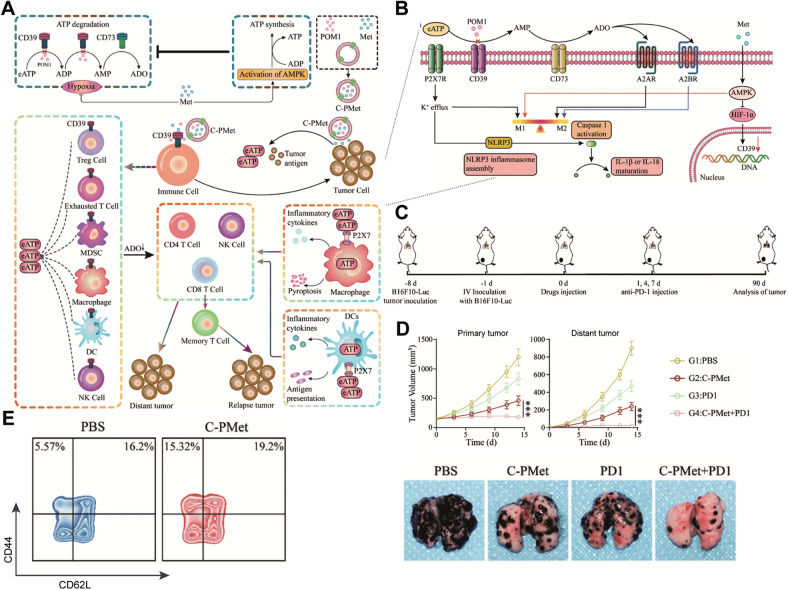
Extracellular ATP-driven antitumor immunity remodels energy metabolism and triggers macrophage pyroptosis. **A** Schematic illustration of antitumor immune responses induced by C-PMet-based immunometabolic therapy. **B** Schematic illustration of immune regulation induced by POM1 and metformin. **C** Schematic illustration of the therapeutic schedule to inhibit tumor lung metastasis. **D** Primary and distant tumor growth curves after the indicated treatments and representative lung photographs. **E** Flow cytometric analysis of memory T cells (CD44^high^CD62^low^, gating on CD3^+^ CD8^+^ T cells) in the spleen. Adapted with permission from [115]; copyright 2022, John Wiley & Sons, Inc.

It is now a matter of urgency to determine how to effectively use novel EV-mediated pyroptosis technology to develop new antitumor immunity schemes, improve specificity and efficiency, reduce chemotherapy resistance, and ensure safety [104]. For example, a recent study employed cancer cell-based exosomes to supply metformin (an AMPK agonist) and POM1 (a CD39 antagonist) for targeted cancer treatment. Targeting the ATP-adenosine pathway via pharmacologically blocked of CD39 and activation of AMPK facilitates the accumulation of pro-inflammatory extracellular ATP (eATP) and reduces immunosuppressive adenosine levels. High eATP levels induce the P2X7-NLRP3 inflammasome to cause macrophage pyroptosis, which potentiates the antigen capacity and maturation of DCs to improve the cytotoxic function of natural killer cells and T cells. C-PMet-mediated immunometabolic treatment can elicit synergistic antitumor immune responses to suppress cancer progression, metastasis, and recurrence and eventually overcome anti-PD1 resistance [115] (Fig. 4). Overall, it is an innovative strategy to promote the application of eATP-dependent antitumor immunity in cancer treatment.

Due to their high biocompatibility and strong bioactivity, sEVs act as a promising tool for therapeutic delivery strategies in cancer therapy. sEV-mediated delivery shows enhanced capacity to penetrate across biological barriers and through tumor blood vessels to accumulate at tumor sites, which greatly improves their therapeutic efficacy. A variety of compounds or drugs have been combined with EVs to develop efficient targeted cancer cell death treatments in numerous preclinical studies. Therefore, modified, engineered, and designer EVs represent a new development trend in this field, and these EVs could be of great clinical value if optimized and integrated properly.

## Conclusions and perspectives

Nonapoptotic RCD forms, mainly ferroptosis, pyroptosis, and necroptosis, have gained increasing attention over the last decade. Understanding the functions of EV-mediated cell death is greatly conducive to providing a favorable technique for the diagnosis and treatment of human cancers. This review outlines the characteristics of EV-mediated non-apoptotic RCD forms and the mechanisms by which EVs transfer different cargoes to mediate tumor progression. Moreover, considering the improved biocompatibility and intrinsic targeting ability of EVs over free drugs, EV-based strategies for targeting cell death regulators shows significant potential with regard to cancer therapy. Accumulating preclinical studies on EV-mediated nonapoptotic RCD forms in cancers provide novel insights into cancer biology and pave the way for the clinical development of EV-based therapeutics.

Recent advances in the expanding roles of EV-mediated cell death hold great potential for both diagnostic and therapeutic applications for cancers. However, several crucial questions remain unanswered: (i) Can the transport of bioactive molecules involved in RCD forms by sEVs in bodily fluids serve as a novel source of candidate biomarkers for cancer diagnosis and prognosis prediction? Altered ferroptosis markers in exosomes have been demonstrated to be promising and easily accessible markers for early cancer detection and prognosis in different cancers, such as HCC and PDAC [116, 117]. Encouragingly, technical progress has led to the development of multimolecular detection platforms, which may promote the development of early cancer detection and treatment monitoring by integrating EV biomarkers [118–120]. (ii) Can nonapoptotic RCD triggered by sEV-based drug delivery systems be harnessed as a novel treatment strategy in cancer therapy? The ineffective delivery of a therapeutic drugs is a major obstacle in the treatment of different cancers. Exosomes have the benefit of high biocompatibility, low immunogenicity, high efficiency, and strong potential for drug delivery. Moreover, erastin@FA-exo can increase the erastin uptake efficiency into MDA‐MB‐231 cells, and ferroptosis stimulated by suppression of System X_c_^−^ has a more pronounced inhibitory effect on the migration and proliferation of breast cancer [108]. Exosome nanovesicles loaded with drugs achieve significant abscopal effects to elicit ATP-mediated antitumor immunity by triggering pyroptosis in macrophages, enhancing the maturation of DCs, and inhibiting tumor-distant metastases [115]. EV-based drug delivery systems could be developed to efficiently deliver antitumor drugs and cell death inducers, thus synergistically suppressing tumor growth. (iii) Can the utilization of sEVs to transport multiple molecules and drugs that mediate cell death serve as an innovative therapeutic approach for use in preclinical studies for cancer treatment? These questions underscore the potential for harnessing the capabilities of sEV-based strategies to improve both cancer diagnosis and treatment. A recent study uncovered a promising triple therapeutic strategy based on inhibiting tumor cell-derived exosome secretion to enhance the effects of ferroptosis-dependent cancer treatment and cancer immunotherapy [121]. Therefore, combination therapies that target multiple cell death pathways while improving therapeutic efficiency should be considered in the future.

Although some research progress has been made regarding the roles of exosome-mediated nonapoptotic RCD forms in tumor progression, most studies were conducted in preclinical models. However, there is encouraging news on the horizon, with certain clinical trials evaluating the impact of key players involved in nonapoptotic RCDs in the context of solid metastatic cancer and hematological malignancies (NCT05493800, NCT04739618). This development emphasizes the growing interest in translating preclinical findings into clinical applications. Consequently, further exploration into the mechanisms associated with EV biogenesis and transport, as well as those related to EV-mediated cell death signaling mechanisms, may have the potential to enhance therapeutic safety and selectivity. This could aid in the development of more effective and targeted treatments for cancer.
